# Non-traditional CD4+CD25−CD69+ regulatory T cells are correlated to leukemia relapse after allogeneic hematopoietic stem cell transplantation

**DOI:** 10.1186/1479-5876-12-187

**Published:** 2014-07-01

**Authors:** Xiao-su Zhao, Xu-hua Wang, Xiang-yu Zhao, Ying-jun Chang, Lan-ping Xu, Xiao-hui Zhang, Xiao-jun Huang

**Affiliations:** 1 Peking University People’s Hospital, Peking University Institute of Hematology, Beijing Key Laboratory of Hematopoietic Stem Cell Transplantation, Beijing 100044, China; 2 Peking-Tsinghua Center for Life Sciences, Beijing 100871, China

**Keywords:** Regulatory T cells, Allogeneic Hematopoietic Stem Cell Transplantation, Relapse, Leukemia, Immunoregulatory

## Abstract

**Background:**

Non-traditional CD4+CD25–CD69+ T cells were found to be involved in disease progression in tumor-bearing mouse models and cancer patients recently. We attempted to define whether this subset of T cells were related to leukemia relapse after allogeneic hematopoietic cell transplantation (allo-HSCT).

**Methods:**

The frequency of CD4+CD25–CD69+ T cells among the CD4+ T cell population from the bone marrow of relapsed patients, patients with positive minimal residual disease (MRD+) and healthy donors was examined by flow cytometry. The CD4+CD25-CD69+ T cells were also stained with the intracellular markers to determine the cytokine (TGF-β, IL-2 and IL-10) secretion.

**Results:**

The results showed that the frequency of CD4+CD25–CD69 + T cells was markedly increased in patients in the relapsed group and the MRD + group compared to the healthy donor group. The percentage of this subset of T cells was significantly decreased after effective intervention treatment. We also analyzed the reconstitution of CD4+CD25–CD69+ T cells at various time points after allo-HSCT, and the results showed that this subset of T cells reconstituted rapidly and reached a relatively higher level at +60 d in patients compared to controls. The incidence of either MRD+ or relapse in patients with a high frequency of CD4+CD25-CD69+ T cells (>7%) was significantly higher than that of patients with a low frequency of CD4+CD25-CD69+ T cells at +60 d, +90 d and +270 d after transplant. However, our preliminary data indicated that CD4+CD25-CD69+ T cells may not exert immunoregulatory function via cytokine secretion.

**Conclusions:**

This study provides the first clinical evidence of a correlation between non-traditional CD4+CD25-CD69+ Tregs and leukemia relapse after allo-HSCT and suggests that exploration of new methods of adoptive immunotherapy may be beneficial. Further research related to regulatory mechanism behind this phenomenon would be necessary.

## Introduction

Allogeneic hematopoietic stem cell transplantation (allo-HSCT) is a potentially curative procedure for patients with hematological malignancies, although leukemia relapse remains one of the most common causes of treatment failure after transplant. However, the precise biological mechanism responsible for relapse remains unclear. Allo-HSCT can also be considered a type of immunotherapy directed toward malignant hematologic diseases or the graft-versus-leukemia (GVL) effect due to either infused immunocytes in the graft or reconstituted T cells after transplant. Thus, it would be beneficial to identify particular subsets of T cells related to the GVL effect as well as graft-versus-host disease (GVHD). Furthermore, confirmation of any correlation between clinical relapse and a specific subset of T cells, as well as further elucidation of the mechanism behind this phenomenon, would be beneficial for exploring new methods of adoptive immunotherapy and preventive strategies against post-transplant relapse.

There is mounting evidence suggesting that regulatory T cells (Tregs) play an important role in maintaining immune homeostasis and regulating negative immune responses [1,2]. It has been shown that Tregs possess the capacity to suppress T cell-mediated GVHD after allo-HSCT [3-7]. However, several studies have demonstrated that the presence of Tregs in either the tumor microenvironment or circulation might be involved in immune escape mechanisms and the failure of the host to trigger an efficient immunological antitumor response [8-10]. Currently, 3 major subtypes of CD4+ Tregs have been recognized to negatively regulate the immune response: CD4+CD25+Foxp3+ cells, Tr1 cells and Th3 cells [11-13]. However, the ineffectiveness of treatment towards CD4+CD25- Tregs in cancer therapy indicates that there may be other mechanisms responsible for the ability of tumor cells to evade immunological attack. Recently, a new subset of CD4+ Tregs that primarily express CD4 and CD69, but not CD25, has been identified in a tumor-bearing mouse model [14]. Han et al. demonstrated that this new type of Tregs could exert immunosuppressive activity via cell-cell contact with membrane-bound TGF-β1 in mice [14]. Another study confirmed that an increase in the number of CD4+CD25-CD69+ Tregs among both the peripheral blood and liver-infiltrating lymphocytes of hepatocellular carcinoma (HCC) patients were correlated with tumor size, vascular invasion and disease stage [15].

Based on these reports, our colleagues demonstrated that a high frequency and increased number of CD4+CD25-CD69+ T cells in the peripheral blood were associated with a reduced risk of acute GVHD. Additionally, a low percentage of this subset of T cells in allografts was shown to predict an increased risk of acute GVHD [16]. These data suggested immunomodulatory effects of these cells and led us to investigate the possible functional roles of CD4+CD25-CD69+ T cells in leukemia relapse after transplantation. Until now, there have been no reports on the immune regulatory action of this subset of Tregs in the relapse of malignant hematological disease. Therefore, we sought to assess the clinical correlation between the number of CD4+CD25-CD69+ T cells in donor bone marrow and leukemia relapse after allo-HSCT, and we also performed a preliminary exploration into the potential immune mechanism responsible for this association.

## Methods

### Patients

Twenty-nine patients with malignant hematological disease who were treated with non-T-cell-depleted allo-HSCT at the Peking University Institute of Hematology from November 2009 to April 2011, including patients undergoing hematological relapse (n = 22) and those with detectable minimal residual disease (MRD, n = 7), were selected as the initial subjects. The remaining 56 patients who received allo-HSCT from October 2009 to July 2010 were also enrolled in this study for the prospective cohort analysis. This study was approved by the ethics committee of Peking University People’s Hospital. Written informed consent was obtained from all patients prior to their entry into the study in accordance with the Declaration of Helsinki. The patient and transplant characteristics are summarized in Additional file 1: Table S1 & Table 1.

**Table 1:** The clinical characteristics of the other 56 patients received allo-HSCT

**Characteristics**	**Total**
Number	56
Patient age, median (range) age	31 (8–54)
Patient sex, male, n (%)	31 (55.0%)
Patient/donor HLA incompatibility	
Related matched	16
Related mis-matched	40
Diagnosis	
AML,n (%)	29
CML,n (%)	1
B-ALL,n (%)	24
MDS,n (%)	1
BAL,n (%)	1
Disease status	
High risk	29 (52.0%)
Standard risk	27 (48.0%)
Situation after allo-HSCT	
No relapse indication	42
MRD+	7
Haematological relapse	5
Extramedullary relapse	2

AML, acute myeloid leukemia; B-ALL, B cell acute lymphoblastic leukemia; CML, chronic myeloid leukemia; MDS, Myelodysplastic syndrome; BAL, biphenotypic acute leukemia.

### Transplant protocols

All of the patients in this study received myeloablative conditioning regimens. Transplantations were performed as previously described [17,18]. Patients who received human leukocyte antigen (HLA)-matched related transplants were treated with either the combination of busulfan (BU, 0.8 mg/kg iv, q6h) and cyclophosphamide (CTX, 1.8 g/m^2^/d for 2 d) or total body irradiation (TBI, 7.7 Gy), given as 1 fraction, followed by CTX. Patients who received related HLA-mismatched transplants were conditioned with either BU + CTX + human antithymocyte globulin (ATG, Sang Stat, Lyon, France) (2.5 mg/kg/d iv for 4 d) or TBI + CTX + ATG. All patients received G-CSF-mobilized bone marrow (BM) and peripheral blood stem cell transfusion followed by cyclosporine (CSA), mycophenolate mofetil (MMF) and short-term methotrexate (MTX). CSA was started on −9 d at a dosage of 2.5 mg/kg iv. The dosage of CSA was adjusted to the blood concentration of 150–250 ng/ml and was gradually reduced and eventually discontinued approximately 4 to 6 months after HSCT. MTX (15 mg/m^2^) was administered by iv on +1 d; patients undergoing related mismatched HSCT were treated with 10 mg/m^2^ MTX on +3 d, +5 d and +11 d, whereas patients undergoing related matched HSCT were treated with 10 mg/m^2^ MTX on +3 d and +6 d.

### Sample collection

Bone marrow from the initial 29 subjects was collected either upon hematological relapse or the detection of MRD. Among these patients, the bone marrow samples of 19 patients who received interventions were also collected. The number of days after the last stem cell infusion is marked with a ‘+’. The MRD status of the 56 patients enrolled in the prospective cohort study was examined at the following time points: +30 d, +60 d, +90 d, +180 d, +270 d and +360 d. Bone marrow samples from patients at the aforementioned time points were also prospectively collected for MRD examination and to determine the number of CD4+CD25-CD69+ cells by flow cytometry (FCM).

### Antibodies and FCM

Bone marrow samples were stained with anti-CD4 peridinin chlorophyll protein (PerCP), anti-CD25 phycoerythrin (PE), anti-CD69 allophycocyanin (APC), anti-CD122 PE and isotype-matched mouse antibodies (BD Biosciences, Mountain View, CA, USA). Flow cytometry was performed using a BD FACSSort machine (Becton Dickinson Biosciences, San Jose, CA, USA). The frequency of CD4+CD25-CD69+ T cells was calculated as the percentage of positive cells in the CD4+ T cell fraction and is shown as the median (range, 25th-75th percentiles). The data were analyzed using CellQuest software (BD Biosciences).

### Definitions

Neutrophil recovery was defined as a neutrophil count >0.5 × 10^9^/l for 3 consecutive days, and platelet recovery was defined as a platelet count >20 × 10^9^/l without transfusions for 7 consecutive days. Patients with malignancies were categorized as ‘standard risk’ if they were either in the first or second complete remission (CR1 or CR2) for acute leukemia or in the chronic phase of chronic myeloid leukemia (CML). Patients were classified as ‘high risk’ if they were past the CR2 phase of acute leukemia, not in remission, Philadelphia chromosome (Ph)-positive, beyond the first chronic phase of CML or myelodysplastic syndrome with refractory anemia with excess of blasts (MDS-RAEB). Hematological relapse was defined as either the reappearance of blasts in the peripheral blood or an unattained CR following a course of standard antileukemia therapy, in which BM was infiltrated with >5% but <20% blasts in a representative smear. The positive MRD (MRD+) used in this study was defined based on the detection of abnormal expression of leukemia-specific genes such as BCR/ABL, AML1/ETO, CBFβ/MYH11, E2A-PBX1 and WT1 and/or leukemia-associated aberrant immune phenotypes (LAIPs). Patients with specific leukemia fusion genes, including AML1/ETO or CBFβ/MYH11, over one log rising, or with other detectable fusion genes, including E2A-PBX1, MLL/AF4 and TLS-ERG, were defined as MRD+. For those patients without leukemia-specific genes, detection of MRD was performed as previously reported [19].

### Isolation and purification of CD4 + CD25-CD69+ T cells

Bone marrow mononuclear cells (BMNC) were isolated from freshly obtained, heparinized bone marrow samples (10 ml) by Ficoll-Hypaque density gradient (1.077 g/dl) centrifugation at 1,500 rpm for 15 minutes. After washing twice, the cells were resuspended in magnetic-activated cell sorting (MACS) buffer (0.5% FBS, 2 mM EDTA in PBS, pH = 7.2). First, CD4+ T cells were negatively selected using the CD4+ T Cell Isolation Kit (Miltenyi Biotec, Auburn, CA; Order no. 130096533) according to the manufacturer’s protocol. The purity of each population was confirmed by FCM to be >95%. Subsequently, the selected CD4+ T cells were incubated with CD25 MicroBeads II (Miltenyi Biotec, Auburn, CA; Order no. 130092983), and CD4+CD25- T cells were isolated according to the manufacturer’s protocol. The purity of each population was confirmed by FCM to be >90%. Finally, the CD69 MicroBead Kit II was used to isolate CD69+ cells from the CD4+CD25- T cells. The isolated cells were stained with anti-CD69 APC antibody to test the purity of the CD4+CD25-CD69+ T cells through FCM analysis.

### Cell surface phenotyping and intracellular staining by flow cytometry

Anti-TGF-β PE, anti-IL-2 PE, anti-IL-10 PE, the other fluorescent antibodies mentioned above and the respective isotype controls were added to BMNCs at 1 × 10^6^/l and incubated for 30 min at 4°C in the dark. The cells were washed once with ice-cold PBS (pH 7.2) containing 0.1% NaN_3_ and 0.5% BSA and resuspended in 200 μl PBS. For intracellular staining, the cells were fixed and permeabilized for 30 min using the eBioscience Staining Intracellular Antigens for Flow Cytometry Kit (eBioscience, San Diego, CA, USA). The cells were washed with permeabilization buffer and labeled with cytokine-specific fluorescence-conjugated anti-IL-2, anti-IL-10 or anti-TGF-β antibodies or the respective isotype-matched Ig controls. The other BMNCs were placed in Iscove’s modified Dulbecco’s medium (IMDM) and incubated with phorbol myristate acetate (PMA) (100 ng/ml) plus ionomycin (1.0 μg/ml, all reagents from Sigma Chemical) for 5 h to stimulate maximum cytokine production; Golgistop (0.7 μl/ml, BD Pharmingen) was added to the samples during the last 4 h to trap the protein in the cytoplasm.

### Statistical analysis

Statistical analyses were performed using SPSS 18.0 (IBM Corporation, Armonk, NY, USA). The data were summarized as median values (range, 25th-75th percentiles). The Mann–Whitney *U* test was used for independent sample comparisons between different groups. For 2-related-sample comparisons of continuous variables, a 2-sided Wilcoxon rank-sum test was performed. The Chi-square test was used for categorical variables. All stated *P*-values are 2-sided, with *P* < 0.05 defined as statistically significant.

## Results

### Higher frequency of CD4+CD25-CD69+ T cells in the bone marrow of MRD + and relapsed patients

**Figure 1:**
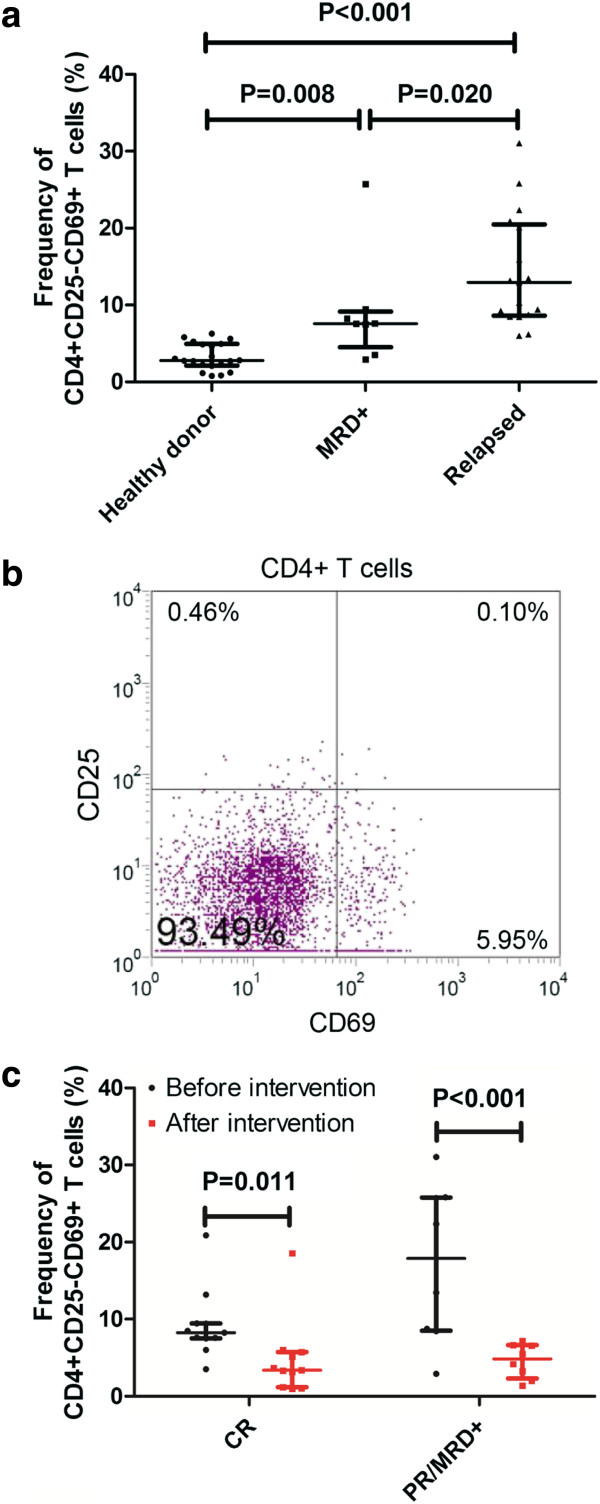
**The frequency of CD4+CD25-CD69 + T cells in bone marrow. (a)** Scatter plot showing the frequency of CD4+CD25-CD69 + T cells (median, range, 25th and 75th percentiles) in healthy donor (n = 20), MRD + patients (n = 7), relapsed patients (n = 22). **(b)** Representative dot plot showing CD69 and CD25 expression on a gated population of CD4+ T cells in a patient. The right figure shows the expression of CD122 on CD4+CD25-CD69+ cells. **(c)** Pooled data showing a comparison of the frequency of CD4+CD25–CD69+T cells (median, range, 25th and 75th percentiles) before and after intervention in patients with different treatment response.

To investigate the relationship between CD4+CD25-CD69+ T cells and leukemia relapse, we first examined the frequency of these cells in the bone marrow from 29 patients who were treated for a malignant hematological disease with allo-HSCT, including patients undergoing hematological relapse (n = 22) and those with a positive MRD status (n = 7). The bone marrow from 20 healthy donors was used as the normal control. The frequency of CD4+CD25-CD69+ T cells in the bone marrow from the healthy donors was 2.79% (range, 2.11-4.94%); however, the frequency of this subset was significantly increased in patients with detectable MRD (7.60%, range, 4.53-9.14%, *P* = 0.008) and those undergoing hematological relapse (12.96%, range, 8.62-20.49%, *P* < 0.001) compared to the control group. Additionally, there was a significant difference in the percentage of CD4+CD25-CD69+ T cells between the relapsed group and MRD + group (*P* = 0.020, Figure 1a). CD69 and CD25 expression on CD4+ T cells in the bone marrow from a representative patient is shown in Figure 1b, demonstrating that this set of T cells highly expresses CD122, as previously reported [14].

Among these 29 patients, bone marrow samples from 19 patients after receiving intervention treatment [including chemotherapy and/or donor lymphocyte infusion (DLI, n = 16) or a second allo-HSCT (n = 3)] were also collected to investigate the correlation of this subset of T cells with treatment response. Eleven patients achieved CR without any detectable MRD, and the other 8 patients either achieved partial remission (PR) or still had detectable MRD. It was also observed that the frequency of CD4+CD25-CD69+ T cells was decreased in both sets of patients after the intervention [CR set, 8.24% (range, 7.53-9.44%) vs. 3.37% (range, 1.17-5.74%), *P* = 0.011; PR/MRD + set, 17.90% (range, 8.52-25.78%) vs. 4.84% (range, 2.32-6.635%), *P* < 0.001, Figure 1c].

### Dynamic monitoring of CD4+CD25-CD69 + T cells in the bone marrow of patients after allo-HSCT

To investigate the dynamic reconstitution of CD4+CD25-CD69+ T cells, the bone marrow from an additional 56 patients at +30 d, +60 d, +90 d, +180 d, +270 d and +360 d after allo-HSCT was collected prospectively and analyzed through FCM. The median follow-up was 628 d (range 107–900 d). The bone marrow samples from the first 3 time points (+30 d, +60 d and +90 d) were available from all of the patients. The samples at +180 d were obtained from 50 patients, the samples at +270 d were obtained from 44 patients, and the samples at +360 d were obtained from 41 patients. All 56 patients achieved hematopoietic reconstitution. During the follow-up, 7 patients (12.5%) experienced hematological or extramedullary relapse at +90 d (range +30-180 d), and 7 patients (12.5%) met the criteria for positive MRD at +90 d (range +30-270 d). Among the relapsed patients, only 1 patient with extramedullary relapse acquired CR after treatment, and the others all died of relapse. Among patients with detectable MRD, 5 achieved CR after intervention, and 2 patients died (one of hematological relapse, the other of DLI-related severe pneumonia). The detailed characteristics of the patients with relapse indications are shown in Additional file 1: Table S2. Forty-two patients did not undergo relapse or had detectable MRD, and only 1 patient died of heart failure during the follow-up period.

**Figure 2:**
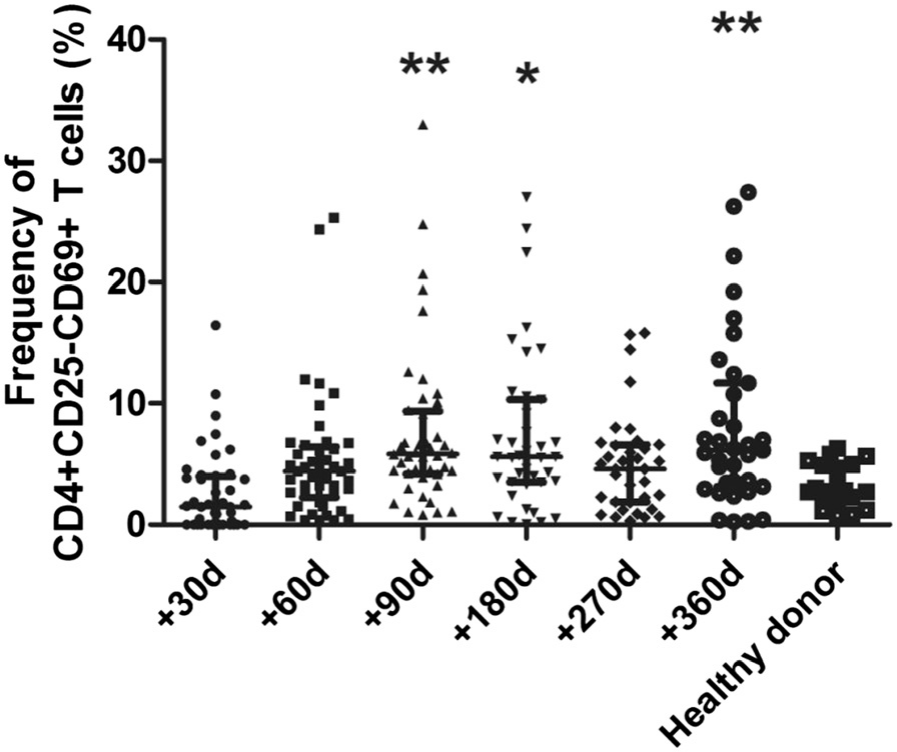
**Dynamic monitoring of CD4+CD25-CD69 + T cells in bone marrow of patients after allo-HSCT.** Scatter plot showing the percentage of CD4+CD25-CD69 + T cells in CD4+ T cells (median, range, 25th and 75th percentiles) in bone marrow of patients without any relapse indication. Compared to healthy donor, **,P < 0.01, *,P < 0.05.

The percentage of CD4+CD25-CD69+ T cells in the bone marrow of 42 patients without any indication of relapse at +30 d (1.49%, range, 0–3.99%), +60 d (4.40%, range, 2.24-6.41%), +90 d (5.82%, range, 4.20-9.34%), +180 d (5.58%, range, 3.53-10.30%), +270 d (4.58%, range, 1.87-6.58%) and +360 d (6.13%, range, 3.14-11.7%) is shown in Figure 2. Although there were no significant differences between the frequency at +30 d, +60 d at +270 d compared to the healthy controls (*P* = 0.390, *P* = 0.099 and *P* = 0.073, respectively, Figure 2), it appeared that the frequencies of CD4+CD25-CD69+ T cells at the other time points were higher than those of the healthy controls, with the exception of +30 d. The percentage of CD4+CD25-CD69+ T cells in patients without any relapse indication at +90 d, +180 d and +360 d was significantly higher than that of the control group (*P* = 0.005, *P* = 0.01 and *P* = 0.004, respectively). These results show that this subset of T cells reconstituted rapidly and reached a relatively higher level at +60 d in patients compared to controls.

### The relationship between relapse and the frequency of CD4+CD25-CD69+ T cells at various time points post-transplantation

We next compared the frequency of CD4+CD25-CD69+ T cells between groups of patients with (n = 14) and without leukemia relapse or relapse indication (n = 42) at each time point after transplantation. The percentages of this subset of T cells in patients undergoing relapse, with an indication for relapse, without any relapse and without an indication for relapse are shown in Table 2. Although there was a significant difference in the frequency of CD4+CD25-CD69+ T cells from the bone marrow at +60 d (*P* = 0.046; *P* > 0.05 at all other time points, Table 2), it appeared that the frequencies of this subset were slightly higher than those in patients without leukemia relapse or any indication of relapse.

**Table 2:** The frequency of CD4+CD25-CD69+ T cells in patients with and without leukemia relapse or relapse indication

**Time point**	**Patients without leukemia relapse or relapse indication Median (range) %**	**Patients with leukemia relapse or relapse indication Median (range) %**	***P *** **value**
+30d	1.49 (0–3.99)	2.08 (0.82-6.87)	0.337
+60d	4.40 (2.24-6.41)	5.94 (4.36-7.75)	0.046
+90d	5.82 (4.20-9.34)	10.09 (3.42-16.70)	0.198
+180d	5.58 (3.53-10.30)	9.87 (4.27-10.92)	0.275
+270d	4.58 (1.87-6.58)	9.04 (3.49-15.56)	0.200
+360d	6.13 (3.14-11.7)	7.03 (4.43-10.04)	0.374

**Figure 3:**
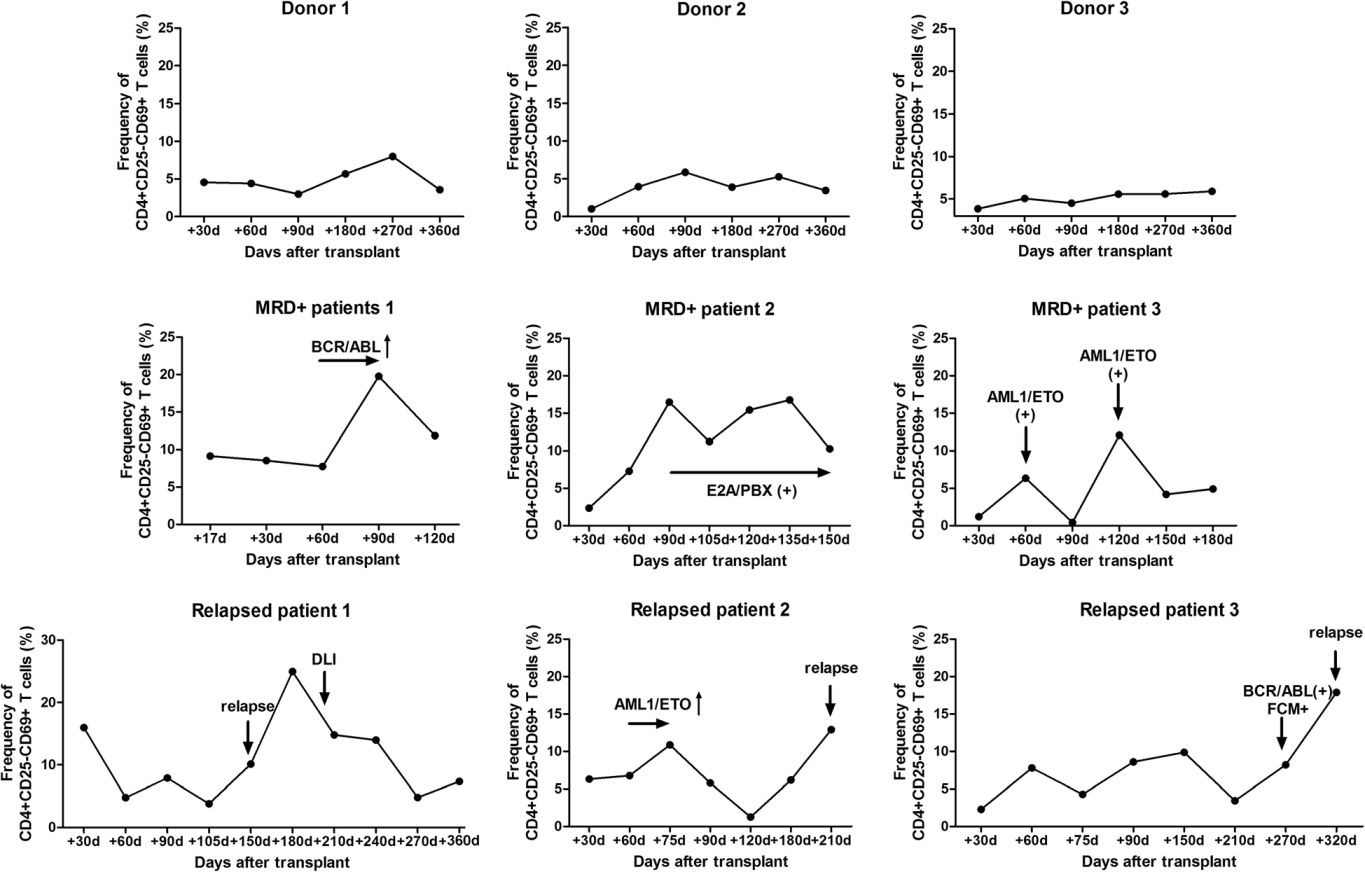
**The dynamic change of frequency of CD4+CD25-CD69+ T cells in the representative patients of different groups (healthy donor, n = 3; patients with MRD + n = 3; relapsed patients, n = 3).** FCM + represents detectable LAIP. DLI, donor lymphocyte infusion.

These 56 patients were then separated into 2 groups at each time point according to the frequency of CD4+CD25-CD69+ T cells. The cutoff value was defined at 7% because it was the upper limit of the CD4+CD25-CD69+ T cell frequency in healthy controls. Patients with a frequency less than 7% were defined as the low CD4+CD25-CD69+ T cell group, whereas the remaining patients were defined as the high CD4+CD25-CD69+ T cell group. The incidence of either MRD + or relapse between these 2 groups was compared at each time point after transplantation. The data in Table 3 show that the incidence of either MRD + or relapse in the high CD4+CD25-CD69+ T cell group was significantly greater than that in the low CD4+CD25-CD69+ T cell group at +60 d, +90 d and +270 d (*P* = 0.012, *P* = 0.027 and *P* = 0.016, respectively). At other time points (+30 d, +180 d and +360 d), there were no significant differences between these 2 groups (*P* > 0.05), but the ratio of patients with either positive MRD or relapse in the high-level group also appeared to be slightly higher than that of the low-level group (Table 4). Figure 3 shows the dynamic change in the frequency of CD4+CD25-CD69+ T cells in representative patients from the different groups (healthy donor, patients with MRD + and relapsed patients).

**Table 3:** Cytokine secretion of CD4+CD25-CD69+ T cells before and after stimulation in vitro

**Cytokine**	**Stimulation PMA & ionomycin**	**Group A (%)**	***P *** **value**	**Group B (%)**	***P *** **value**
IL-2	Before	6.4 (1.4-13.0)	0.347	6.3 (4.3-9.2)	0.917
	After	9.3 (1.8-18.4)		6.0 (4.0-10.9)	
IL-10	Before	1.5 (0–7.8)	1.000	4.1 (0.3-12.0)	0.754
	After	3.2 (0–7.8)		4.8 (1.9-14.2)	
TGFβ	Before	3.7 (3.2-13.6)	0.117	7.9 (5.7-14.2)	0.602
	After	6.5 (5.3-23.5)		6.9 (5.0-20.7)	

Data represent the median% (range) of specific cytokine-secreted CD4+CD25-CD69+ T cells.

**Table 4:** The incidence of MRD + or relapse comparison between high CD4+CD25-CD69+ T cells group and low CD4+CD25-CD69+ T cells group at different time point after allo-HSCT

**Time point**	**Total samples (n)**	**MRD + or relapse in low level groups**	**MRD + or relapse in high level groups**	***P *** **value**
+30d	56	11/49	3/7	0.350
+60d	56	8/45	6/11	0.012
+90d	56	5/34	9/22	0.027
+180d	50	8/31	6/19	0.175
+270d	44	3/33	5/11	0.016
+360d	41	3/25	3/16	0.662

The percentage less than 7% was defined as low level group, otherwise was defined as high level group.

### CD4+CD25-CD69+ T cells may not display immunoregulatory function via cytokine secretion

**Figure 4:**
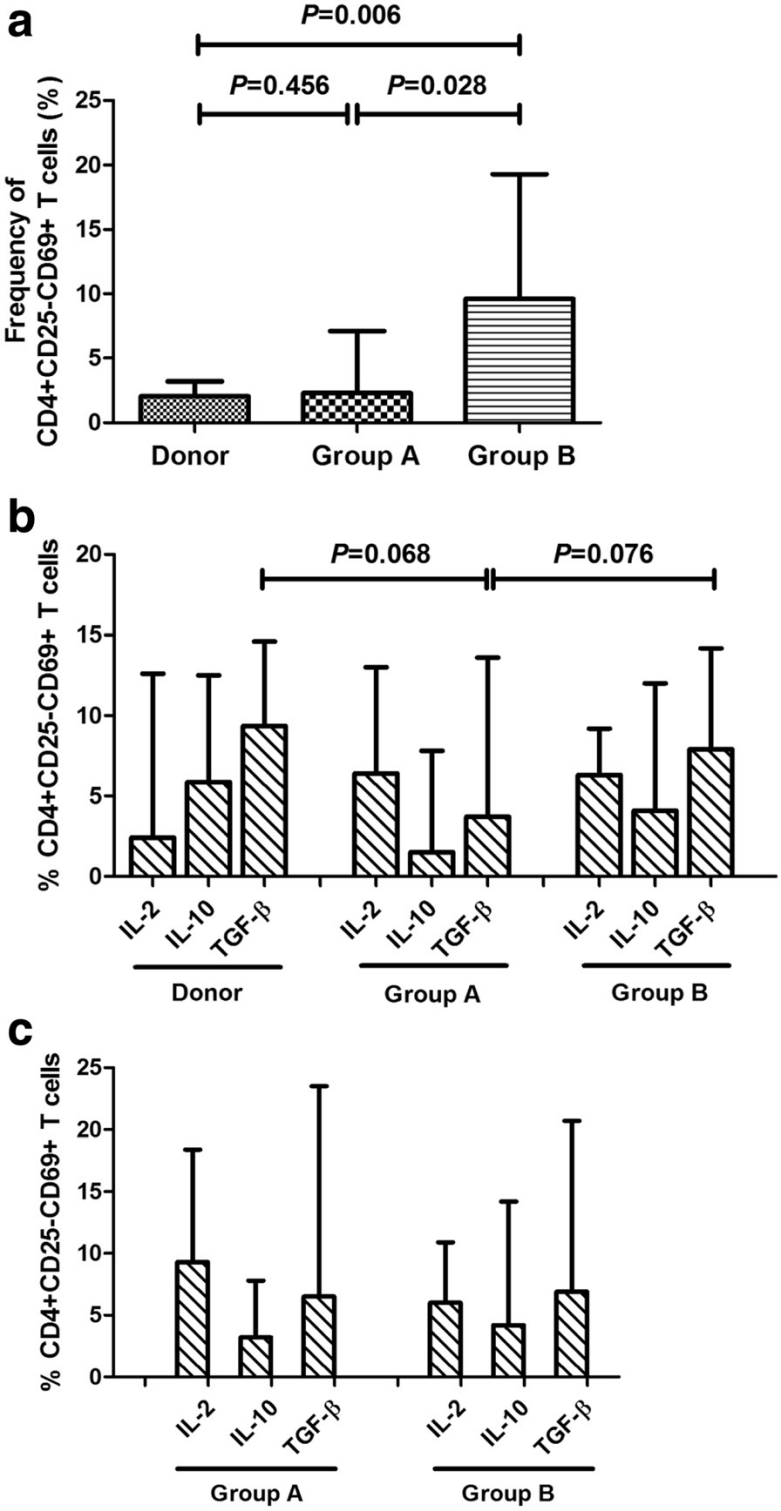
**CD4+CD25-CD69+ T cells might not display immune regulatory function via cytokine secretion. (a)**. The frequency of CD4+CD25-CD69+ T cells in group B was significantly higher than those in group A and healthy donors. **(b)**. No significant difference of cytokine (IL-2, IL-10 and TGF-β) level was seen among donors and patients of group A & B before PMA and ionomycin stimulation. **(c)**. There was no obvious difference of cytokine secretion between group A and group B after PMA and ionomycin stimulation.

The results from the clinical analysis suggested that these non-traditional CD4+CD25–CD69+ Tregs were correlated with leukemia relapse after allo-HSCT. Thus, we attempted to confirm the immunoregulatory roles of CD4+CD25-CD69+ T cells in vitro. First, we attempted to isolate this subset of T cell from bone marrow using specific T cell isolation kits. However, due to the 3-marker phenotype and low frequency of these cells, the use of 3 types of isolation (including CD4 negative selection, CD25 positive selection and CD69 positive selection) produced too few target cells for the mixed leukocyte reactions (data not shown). We then analyzed the secretion of several important Th1/Th2 cytokines, including IL-2, IL-10 and TGF-β, in this subset of T cells to investigate whether they may be responsible for the proposed immunoregulatory function of this subset. The cytokine secretion of CD4+CD25-CD69+ T cells in unstimulated (PMA and ionomycin) bone marrow was analyzed in healthy donors (n = 6), patients without any relapse indications (n = 5, termed as group A) and MRD + patients or relapsed patients (MRD + patients, n = 3; relapsed patients, n = 2; termed group B). As shown in Figure 4a and in accordance with the results described above, the frequency of CD4+CD25-CD69+ T cells in group B was significantly higher than that in group A (*P* = 0.028) and that in healthy donors (*P* = 0.006). Despite the increased number of CD4+CD25-CD69+ T cells that secreted TGF-β in unstimulated bone marrow from group B compared to group A, this difference was not statistically significant (*P* = 0.068 and *P* = 0.076, respectively, Figure 4b), and no significant differences in IL-2 and IL-10 secretion were observed among these 3 groups (*P* > 0.05, Figure 4b). Because CD69 is generally considered an early activation marker of T cells, we then examined whether the CD4+CD25-CD69+ T cells in patients undergoing allo-HSCT constituted activated T cells. The secretion of IL-2, IL-10 and TGF-β in CD4+CD25-CD69+ T cells from the bone marrow after PMA and ionomycin stimulation was analyzed in patients in groups A and B, although it appeared that the levels of these cytokines were unaffected by PMA and ionomycin stimulation (Table 3). Similar to the results of the unstimulated bone marrow samples, no clear differences in cytokine secretion could be observed between the patients in group A and group B after stimulation (*P* > 0.05, Figure 4c).

## Discussion

Among the main recognized subsets of CD4+ T cells involved in negative regulation of the immune response, most studies have focused on the regulatory function of CD4+CD25 + Foxp3+ Tregs [20]. In addition to these well-characterized CD4+CD25+ Tregs, CD4+CD25- T cells have also been shown to display inhibitory immune function in some animal models [21-23]. This T cell subset was shown to mediate immune tolerance by attenuating the autoimmune response via both cell-cell contact mechanisms and the regulation of soluble factor expression (IL-10 and IFN-γ) [24]. These reports provided new insight into the mechanisms of tolerance to allografted transplant. However, CD4+CD25-CD69+ T cells, which were the subject of our study, not only possessed a unique immunophenotype but also functioned independently of cytokine secretion. Previous studies have demonstrated the immunosuppressive role of CD4+CD25- Tregs in solid tumor models [14,15], but to our knowledge, no study has focused on the functional roles of this newly identified T cell subset in the development of hematological malignant disease as well as immunoregulation after allo-HSCT. Here, we investigated the functional roles of this subset of T cells in patients with hematological malignancy after allo-HSCT. Thus, this is the first study to explore the relationship between CD4+CD25-CD69+ T cells and the progression of hematological tumors post-transplantation. Our results indicate this relationship to be an extension of the immunoregulatory role of these newly identified Tregs beyond their role in solid tumors.

It remains controversial which source of CD4+CD25-CD69+ T cells should be selected as the target for tumor immunity. According to the pathogenesis of acute GVHD, it is likely the CD4+CD25-CD69+ T cells in the peripheral blood that display a significant inhibitory effect during the course of GVHD. Regarding antitumor immunity, previous studies have been performed in tumor-bearing mouse models and patients with HCC (liver-infiltrating lymphocytes), and it is known that tumor cells can recruit and enrich Tregs to suppress antitumor activity [8,10]. Because hematological malignant cells are derived from and primarily localize in the bone marrow, we selected CD4+CD25-CD69+ T cells from the bone marrow as the focus of our study. Indeed, we observed a correlation between leukemia relapse and the frequency of this new type of Treg. Additionally, these T cells were also positively correlated to tumor burden prior to any intervention after transplant. We then sought to examine whether CD4+CD25-CD69+ T cells were directly responsible for relapse or if the presence of a tumor induced the increase in this subset of T cells, which led us to the following question: what is the biological role of CD4+CD25-CD69+ T cells during allo-HSCT?

Our preliminary data demonstrated that the frequencies of these non-traditional Tregs were relatively high compared to those of normal control subjects at each time point post-transplantation except at +30 d. This finding indicated that these CD4+CD25-CD69+ T cells could be reconstituted quickly and even expanded after transplantation. At the early stages after transplant, the low frequency of CD4+CD25-CD69+ T cells may be due to incomplete T cell reconstitution. During the second month, the frequency of these T cells continued to increase and was maintained at a higher level compared to the normal control group. A similar phenomenon has also been reported for other immunoregulatory cells during HSCT. For example, myeloid-derived suppressor cells (MDSCs), which recently have been shown to possess immunosuppressive function, expanded after HSCT both in pre-clinical mouse models and patients with successful transplants [25,26]. In vitro functional experiments had further demonstrated the immunoregulatory role of CD4+CD25-CD69+ T cells in a mouse model [14]. Thus, we speculated that this subset might contribute to the maintenance of immune tolerance after transplantation. Our previous data also identified a negative correlation between the frequency of this subset in the peripheral blood and the incidence of aGVHD; in particular, the lower frequency of this subset of T cells led to more severe aGVHD [16]. To a large extent, this result indicated that CD4+CD25-CD69+ T cells may exert important immunoregulatory functions after transplantation. In this study, we provided the clinical evidence to support such an immunosuppressive effect of CD4+CD25-CD69+ T cells during HSCT. The frequency of this subset of T cells in the bone marrow was significantly increased in relapsed patients, whereas in patients with a lower tumor load (MRD+), a relatively lower proportion of these T cells was observed. However, the percentage of these T cells from patients who either relapsed or had a positive MRD was higher compared to those of normal control subjects and patients with no relapse indication at other time points, with the exception of +30 d. Furthermore, the number of CD4+CD25-CD69+ T cells declined rapidly after intervention. Together, these data indicated that the immunosuppressive effect of this subset of T cells likely contributed to leukemia relapse after transplantation. However, as a type of immunomodulatory cell, their frequency may not directly reflect the load of the malignancy; instead, these cells are more indicative of the immune status of the transplant recipient. Consistent with this theory, distinct clinical outcomes for these patients were obtained despite the significant decrease in the frequency of MRD + or relapsed patients after intervention therapy. For those patients who did not achieve complete remission, it was hypothesized that their leukemia cells possessed a greater likelihood of malignant behavior.

Although the frequency of CD4+CD25-CD69+ T cells could not accurately predict the forthcoming relapse at each time point (with the exception of +60 d, +90 d and +270 d), it appeared that this subset of CD4+ T cells was increased prior to the presence of either MRD + or leukemia relapse, according to this case study. The median time for patients undergoing MRD + or leukemia relapse was +90 d, which may explain why a significant group difference at +60 d after transplant was observed regardless of the perspective. These results confirmed that CD4+CD25-CD69+ T cells are negative immunomodulatory cells. When the frequency of these non-traditional Tregs reaches an excessive level, patients may develop an excessive form of immune tolerance and suffer from an impending relapse. As a result, due to this disruption of the immune balance, the tumor may have a chance to escape immunological surveillance. Therefore, the frequency of CD4+CD25-CD69+ T cells could represent a suitable parameter to predict the incidence of leukemia relapse after transplantation.

There are 3 possibilities that could explain the suboptimal concordance between the frequency of CD4+CD25-CD69+ T cells and leukemia relapse. First, because 14 patients had either relapsed or developed MRD + at various time points post-transplantation, the accuracy of relapse prediction at certain time points could have been impaired. Second, the interval of sample collection may have affected the accuracy of predicting relapse, and the interval between sample collection and relapse could have also interfered with the predictive efficiency. Third, the bone marrow is a hematopoietic organ where many types of hematopoietic cells localize, so there exists the possibility that a small number of activated T cells also expressing CD69 are present at this location. Although the CD4+CD25-CD69+ T cells we detected also highly express CD122, there may be other unknown molecular markers that could discriminate this new regulatory T cell from classically activated T cells.

Finally, to investigate whether the CD4+CD25-CD69+ T cells in the bone marrow were the same subset of T cells that had been examined in previous studies [14,15], we measured the cytokine secretion of these T cells. Consistent with previous reports, this subset of T cell did not respond to PMA/ionomycin stimulation. This finding indicated that despite the difference in the ratio of this non-traditional Treg in relapsed and non-relapsed patients, the cells secreted comparable levels of IL-2, IL-10 and TGF-β. This finding also suggested that these novel Tregs might exert their immunosuppressive function independent of cytokine secretion and through other means such as cell-cell contact.

## Conclusions

In summary, this study presents the first clinical evidence for the positive correlation between non-traditional CD4+CD25-CD69+ Tregs and leukemia relapse after allo-HSCT, although further study is necessary to confirm our findings. Moreover, we demonstrated the immunosuppressive role of this subset of T cell both in the peripheral blood and bone marrow towards GVHD and GVL post-transplantation, respectively. However, the mechanism underlying their immunosuppressive function remains unclear. The precise role of CD4+CD25-CD69+ T cells in leukemia relapse also requires further exploration and may lead to the discovery of new methods of adoptive immunotherapy.

## Competing interests

The authors declare that they have no competing interests.

## Authors’ contributions

XS Zhao carried out the flow cytometry assay, performed the statistical analysis and drafted the manuscript. XH Wang also carried out the flow cytometry assay and participated in data analysis. XJ Huang participated in the design of the study and revised it critically for important intellectual content. All other authors were involved in the study design discussions and treated patients at Peking University Institute of Hematology. All authors read and approved the final manuscript.

## Authors’ information

The current address for all authors and corresponding author:

Peking University Institute of Hematology, Peking University People’s Hospital

No 11 Xizhimen South Street, Beijing 100044, China

## Supplementary Material

Additional file 1: Table S1 The clinical characteristics of 29 patients with hematological relapse or with detectable MRD. **Table S2.** The characteristics of patients among 56 cases who developed into MRD + or relapse after allo-HSCT. Click here for file

## References

[B1] Sakaguchi S Regulatory T cells in the past and for the future Eur J Immunol 2008 38 901 937 10.1002/eji.200890012 18395855

[B2] Sakaguchi S Sakaguchi N Asano M Itoh M Toda M Immunologic self-tolerance maintained by activated T cells expressing IL-2 receptor alpha-chains (CD25). Breakdown of a single mechanism of self-tolerance causes various autoimmune diseases J Immunol 1995 155 1151 1164 7636184

[B3] Brunstein CG Miller JS Cao Q McKenna DH Hippen KL Curtsinger J Defor T Levine BL June CH Rubinstein P McGlave PB Blazar BR Wagner JE Infusion of ex vivo expanded T regulatory cells in adults transplanted with umbilical cord blood: safety profile and detection kinetics Blood 2011 117 1061 1070 10.1182/blood-2010-07-293795 20952687 PMC3035067

[B4] Lokhorst HM Wu K Verdonck LF Laterveer LL van de Donk NW van Oers MH Cornelissen JJ Schattenberg AV The occurrence of graft-versus-host disease is the major predictive factor for response to donor lymphocyte infusions in multiple myeloma Blood 2004 103 4362 4364 10.1182/blood-2003-11-3862 14976044

[B5] Mutis T van Rijn RS Simonetti ER Aarts-Riemens T Emmelot ME van Bloois L Martens A Verdonck LF Ebeling SB Human regulatory T cells control xenogeneic graft-versus-host disease induced by autologous T cells in RAG2−/−gammac−/− immunodeficient mice Clin Cancer Res 2006 12 5520 5525 10.1158/1078-0432.CCR-06-0035 17000688

[B6] Soiffer R Immune modulation and chronic graft-versus-host disease Bone Marrow Transplant 2008 42 Suppl 1 S66 S69 18724307 10.1038/bmt.2008.119

[B7] Tricot G Vesole DH Jagannath S Hilton J Munshi N Barlogie B Graft-versus-myeloma effect: proof of principle Blood 1996 87 1196 1198 8562947

[B8] Rabinovich GA Gabrilovich D Sotomayor EM Immunosuppressive strategies that are mediated by tumor cells Annu Rev Immunol 2007 25 267 296 10.1146/annurev.immunol.25.022106.141609 17134371 PMC2895922

[B9] Wang HY Wang RF Regulatory T cells and cancer Curr Opin Immunol 2007 19 217 223 10.1016/j.coi.2007.02.004 17306521

[B10] Zou W Regulatory T cells, tumour immunity and immunotherapy Nat Rev Immunol 2006 6 295 307 10.1038/nri1806 16557261

[B11] Buckner JH Ziegler SF Regulating the immune system: the induction of regulatory T cells in the periphery Arthritis Res Ther 2004 6 215 222 10.1186/ar1226 15380036 PMC546291

[B12] Ziegler SF FOXP3: of mice and men Annu Rev Immunol 2006 24 209 226 10.1146/annurev.immunol.24.021605.090547 16551248

[B13] Lan RY Ansari AA Lian ZX Gershwin ME Regulatory T cells: development, function and role in autoimmunity Autoimmun Rev 2005 4 351 363 10.1016/j.autrev.2005.01.007 16081026

[B14] Han Y Guo Q Zhang M Chen Z Cao X CD69+ CD4+ CD25- T cells, a new subset of regulatory T cells, suppress T cell proliferation through membrane-bound TGF-beta 1 J Immunol 2009 182 111 120 10.4049/jimmunol.182.1.111 19109141

[B15] Zhu J Feng A Sun J Jiang Z Zhang G Wang K Hu S Qu X Increased CD4(+) CD69(+) CD25(−) T cells in patients with hepatocellular carcinoma are associated with tumor progression J Gastroenterol Hepatol 2011 26 1519 1526 10.1111/j.1440-1746.2011.06765.x 21557772

[B16] Lu SY Huang XJ Liu KY Liu DH Xu LP High frequency of CD4+ CD25–CD69+ T cells is correlated with a low risk of acute graft-versus-host disease in allotransplants Clin Transplant 2012 26 E158 E167 10.1111/j.1399-0012.2012.01630.x 22507356

[B17] Huang XJ Liu DH Liu KY Xu LP Chen H Han W Chen YH Wang JZ Gao ZY Zhang YC Jiang Q Shi HX Lu DP Haploidentical hematopoietic stem cell transplantation without in vitro T-cell depletion for the treatment of hematological malignancies Bone Marrow Transplant 2006 38 291 297 10.1038/sj.bmt.1705445 16883312

[B18] Huang XJ Wang Y Liu DH Xu LP Chen H Chen YH Han W Shi HX Liu KY Modified donor lymphocyte infusion (DLI) for the prophylaxis of leukemia relapse after hematopoietic stem cell transplantation in patients with advanced leukemia–feasibility and safety study J Clin Immunol 2008 28 390 397 10.1007/s10875-008-9193-4 18347959

[B19] Yan CH Liu DH Liu KY Xu LP Liu YR Chen H Han W Wang Y Qin YZ Huang XJ Risk stratification-directed donor lymphocyte infusion could reduce relapse of standard-risk acute leukemia patients after allogeneic hematopoietic stem cell transplantation Blood 2012 119 3256 3262 10.1182/blood-2011-09-380386 22337715

[B20] Colombo MP Piconese S Regulatory-T-cell inhibition versus depletion: the right choice in cancer immunotherapy Nat Rev Cancer 2007 7 880 887 10.1038/nrc2250 17957190

[B21] Feunou P Poulin L Habran C Le Moine A Goldman M Braun MY CD4+CD25+ and CD4+CD25- T cells act respectively as inducer and effector T suppressor cells in superantigen-induced tolerance J Immunol 2003 171 3475 3484 10.4049/jimmunol.171.7.3475 14500643

[B22] Stephens LA Mason D CD25 is a marker for CD4+ thymocytes that prevent autoimmune diabetes in rats, but peripheral T cells with this function are found in both CD25+ and CD25- subpopulations J Immunol 2000 165 3105 3110 10.4049/jimmunol.165.6.3105 10975823

[B23] Graca L Thompson S Lin CY Adams E Cobbold SP Waldmann H Both CD4(+)CD25(+) and CD4(+)CD25(−) regulatory cells mediate dominant transplantation tolerance J Immunol 2002 168 5558 5565 10.4049/jimmunol.168.11.5558 12023351

[B24] Degauque N Lair D Braudeau C Haspot F Sebille F Dupont A Merieau E Brouard S Soulillou JP Development of CD25- regulatory T cells following heart transplantation: evidence for transfer of long-term survival Eur J Immunol 2007 37 147 156 10.1002/eji.200635879 17171754

[B25] Luyckx A Schouppe E Rutgeerts O Lenaerts C Koks C Fevery S Devos T Dierickx D Waer M Van Ginderachter JA Billiau AD Subset characterization of myeloid-derived suppressor cells arising during induction of BM chimerism in mice Bone Marrow Transplant 2012 47 985 992 10.1038/bmt.2011.207 22041852

[B26] Mougiakakos D Jitschin R von Bahr L Poschke I Gary R Sundberg B Gerbitz A Ljungman P Le Blanc K Immunosuppressive CD14+HLA-DRlow/neg IDO + myeloid cells in patients following allogeneic hematopoietic stem cell transplantation Leukemia 2013 27 377 388 10.1038/leu.2012.215 22828446

