# Supplementary material for: Non-traditional CD4+CD25−CD69+ regulatory T cells are correlated to leukemia relapse after allogeneic hematopoietic stem cell transplantation
Source: J Transl Med. 2014 Jul 1;12:187. doi: 10.1186/1479-5876-12-187 (PMC4089938; doi:10.1186/1479-5876-12-187)
Supplement: Additional file 1: Table S1 — The clinical characteristics of 29 patients with hematological relapse or with detectable MRD. Table S2. The characteristics of patients among 56 cases who developed into MRD + or relapse after allo-HSCT. [file 1479-5876-12-187-S1.docx]

Additional file 1: Table S1. The clinical characteristics of 29 patients with hematological relapse or with detectable MRD.

| Patient  number/sex | Age | Original Disease | Patient/donor  HLA compatibility | Diagnosis as relapse or MRD+ | time after alloHSCT |
| --- | --- | --- | --- | --- | --- |
| 1/F | 17 | AML | 4/6 | WT1↑ | 1y10d |
| 2/M | 27 | AML | 3/6 | WT1↑ | 204d |
| 3/M | 41 | B-ALL | 6/6 | WT1↑MRD+ | 1y98d |
| 4/M | 6 | B-ALL | 4/6 | WT1↑ | 223d |
| 5/M | 36 | AML | 6/6 | AML1-ETO↑MRD+ | 30d |
| 6/M | 5 | AML | 6/6 | WT1↑TLS-ERG↑ | 132d |
| 7/M | 27 | AML | 5/6 | WT1↑ | 1y80d |
| 8/F | 34 | B-ALL | 3/6 | 40%Blast | 72d |
| 9/M | 30 | AML | 4/6 | 31%Blast | 2y140d |
| 10/F | 19 | AML | 6/6 | 67%Blast | 107d |
| 11/M | 19 | AML | 3/6 | 34%Blast | 230d |
| 12/F | 46 | AML | ?/6 | 51%Blast | 170d |
| 13/M | 19 | B-ALL | 4/6 | 79%Blast | 150d |
| 14/F | 41 | AML | 4/6 | 14%Blast | 2y22d |
| 15/M | 18 | AML | 4/6 | 35%Blast | 1y76d |
| 16/F | 27 | AML | 4/6 | 50%Blast | 72d；170d |
| 17/M | 17 | AML | 6/6 | 48%Blast | 126d |
| 18/M | 16 | CML | 3/6 | 31%Blast | 188d |
| 19/M | 22 | CML | 3/6 | 44%Blast | 1y295d |
| 20/M | 32 | AML | 4/6 | 59%Blast | 102d |
| 21/M | 16 | B-ALL | 6/6 | 73%Blast | 1y250d |
| 22/F | 24 | B-ALL | 3/6 | 74%Blast | 173d |
| 23/M | 27 | B-ALL | 4/6 | 35%Blast | 134d |
| 24/F | 43 | AML | 4/6 | 34%Blast | 1y29d |
| 25/M | 11 | B-ALL | 4/6 | 88%Blast | 1y338d |
| 26/F | 30 | B-ALL | 3/6 | 20%Blast | 1y153d |
| 27/F | 36 | CML | 3/6 | 23%Blast | 1y68d |
| 28/F | 19 | AML | 3/6 | Left posterior mediastinal tumor | 349d |
| 29/F | 34 | B-ALL | 3/6 | Right brest tumor | 1y57d |

F, female; M, male; AML, acute myeloid leukemia; B-ALL, B cell acute lymphoblastic leukemia; CML, chronic myeloid leukemia; WT1, Wilms Tumor suppressor-1; MRD, minimal residual disease; y, year; d, day

Table S2. The characteristics of patients among 56 cases who developed into MRD+ or relapse after allo-HSCT.

| Patient  number/sex | Age | Original Disease | Patient/donor  HLA compatibility | Diagnosis as MRD+ or relapsed | time after allo-HSCT |
| --- | --- | --- | --- | --- | --- |
| 1/ M | 27 | AML | 3/6 | MRD+(CBFβ-MYH11↑, FCM+) | 30d |
| 9/ F | 35 | B-ALL | 3/6 | MRD+(MLL/AF4↑, FCM+) | 105d |
| 17/ F | 26 | B-ALL | 3/6 | MRD+(E2A-PBX1↑, FCM+) | 90d |
| 20 / M | 44 | AML | 6/6 | MRD+(AML1-ETO↑, FCM+) | 270d |
| 24/ M | 40 | B-ALL | 6/6 | MRD+(BCR-ABL↑, FCM+) | 90d |
| 37/ M | 44 | AML | 6/6 | MRD+(AML1-ETO↑, FCM+) | 30d |
| 56/ M | 40 | AML | 5/6 | MRD+(CBFB-MYH11↑, WT1↑) | 90d |
| 3/ M | 14 | AML | 4/6 | 22%Blast | 120d |
| 10/ M | 18 | AML | 3/6 | 80%Blast | 90d |
| 21/ M | 18 | B-ALL | 5/6 | 72%Blast | 90d |
| 22/ F | 51 | B-ALL | 6/6 | 23%Blast | 30d |
| 23/ M | 26 | AML | 5/6 | 63%Blast | 60d |
| 27/ M | 28 | AML | 3/6 | Granulocytic sarcoma | 30d |
| 48/ F | 47 | AML | 3/6 | Granulocytic sarcoma | 180d |

F, female; M, male; AML, acute myeloid leukemia; ALL: acute lymphoblastic leukemia; CML, chronic myeloid leukemia; y, year; d, day; Blast, blast cell in bone marrow; MRD+, detectable minimal residual disease; FCM+, detectable leukemia-associated aberrant immune phenotypes.
